# (*E*)-1-(2,4,6-Trihy­droxy­benzyl­idene)-4-ethyl­thio­semicarbazide dihydrate

**DOI:** 10.1107/S1600536810030783

**Published:** 2010-08-11

**Authors:** Hana Bashir Shawish, Kong Wai Tan, M. Jamil Maah, Seik Weng Ng

**Affiliations:** a Department of Chemistry, University of Malaya, 50603 Kuala Lumpur, Malaysia

## Abstract

In the title mol­ecule, C_10_H_13_N_3_O_3_S·2H_2_O, the thio­semi­carbazide =N—NH—C(=S)—NH– fragment [torsion angle = 0.2 (1)°] is nearly coplanar with the benzene ring [dihedral angle = 2.4 (1)°]. The benzene ring and semicarbazide moiety are located on opposite sites of the C=N bond, showing an *E* configuration. The hy­droxy, imino and water H atoms are engaged in extensive hydrogen bonding, forming a three-dimensional network.

## Related literature

For the crystal structure of a related compound, 1-(2,3,4-trihy­droxy­benzyl­idene)-4-ethyl­thio­semicarbazide, see: Shaw­ish *et al.* (2010 ▶).
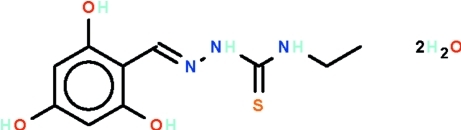


## Experimental

### 

#### Crystal data

C_10_H_13_N_3_O_3_S·2H_2_O
*M* *_r_* = 291.33
Monoclinic, 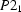

*a* = 4.6645 (4) Å
*b* = 10.4006 (9) Å
*c* = 13.5381 (11) Å
β = 98.674 (1)°
*V* = 649.27 (10) Å^3^
*Z* = 2
Mo *K*α radiation
μ = 0.27 mm^−1^
*T* = 100 K
0.30 × 0.20 × 0.10 mm

#### Data collection

Bruker SMART APEX diffractometer
Absorption correction: multi-scan (*SADABS*; Sheldrick, 1996 ▶) *T*_min_ = 0.923, *T*_max_ = 0.973
6232 measured reflections
2937 independent reflections
2826 reflections with *I* > 2σ(*I*)
*R*_int_ = 0.025

#### Refinement

*R*[*F* ^2^ > 2σ(*F* ^2^)] = 0.028
*wR*(*F* ^2^) = 0.074
*S* = 1.03
2937 reflections
208 parameters
12 restraints
H atoms treated by a mixture of independent and constrained refinement
Δρ_max_ = 0.24 e Å^−3^
Δρ_min_ = −0.19 e Å^−3^
Absolute structure: Flack (1983 ▶), 1370 Friedel pairs
Flack parameter: −0.05 (6)

### 

Data collection: *APEX2* (Bruker, 2009 ▶); cell refinement: *SAINT* (Bruker, 2009 ▶); data reduction: *SAINT*; program(s) used to solve structure: *SHELXS97* (Sheldrick, 2008 ▶); program(s) used to refine structure: *SHELXL97* (Sheldrick, 2008 ▶); molecular graphics: *X-SEED* (Barbour, 2001 ▶); software used to prepare material for publication: *publCIF* (Westrip, 2010 ▶).

## Supplementary Material

Crystal structure: contains datablocks global, I. DOI: 10.1107/S1600536810030783/xu5011sup1.cif

Structure factors: contains datablocks I. DOI: 10.1107/S1600536810030783/xu5011Isup2.hkl

Additional supplementary materials:  crystallographic information; 3D view; checkCIF report

## Figures and Tables

**Table 1:** Hydrogen-bond geometry (Å, °)

*D*—H⋯*A*	*D*—H	H⋯*A*	*D*⋯*A*	*D*—H⋯*A*
O1—H1o⋯N1	0.85 (1)	1.99 (2)	2.722 (2)	144 (2)
O2—H2o⋯O1^i^	0.83 (1)	2.27 (2)	2.955 (2)	140 (2)
O3—H3o⋯O1*W*	0.85 (2)	1.76 (2)	2.598 (2)	174 (2)
O1w—H11⋯O2w	0.84 (1)	1.94 (1)	2.785 (2)	177 (3)
O1w—H12⋯O3^ii^	0.84 (1)	2.22 (1)	3.002 (2)	155 (2)
O2w—H21⋯S1^iii^	0.84 (1)	2.45 (1)	3.279 (1)	169 (2)
O2w—H22⋯S1^iv^	0.84 (1)	2.49 (1)	3.292 (1)	162 (2)
N2—H2n⋯O2w^v^	0.86 (1)	2.13 (1)	2.965 (2)	166 (2)
N3—H3n⋯O2^vi^	0.86 (1)	2.26 (2)	2.934 (2)	136 (2)

Symmetry codes: (i) 
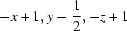
; (ii) 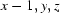; (iii) 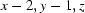; (iv) 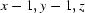; (v) 
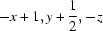
; (vi) 
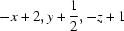
.

**Fig. 1.**
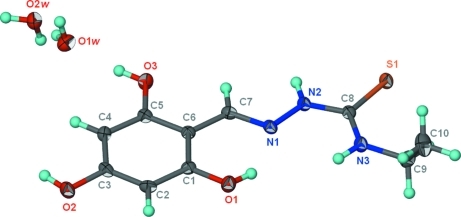
Thermal ellipsoid plot (Barbour, 2001) of C10H13N3O3S.2H2O at the 70% probability level; hydrogen atoms are drawn as spheres of arbitrary radius.

## supplementary crystallographic information

### Experimental

2,4,6-Trihydroxybenzaldehyde (1.54 g, 10 mmol) and 4-ethylthiosemicarbazide (1.19 g, 1 mmol) were heated in ethanol (20 ml) for 2 h; acetic acid (0.5 ml) was also added. A brown solid separated from the cool solution; this was recrystallized from methanol.

### Refinement

Carbon-bound H-atoms were placed in calculated positions (C—H 0.95 to 0.99 Å) and were included in the refinement in the riding model approximation, with *U*(H) set to 1.2 to 1.5*U*(C).

The imino H and hydroxy H atoms were located in a difference Fourier map, and were refined with distance restraints of N–H 0.86±0.01 and O–H 0.84±0.01 Å; their temperature factors were freely refined.

### Crystal data

**Table:**

C_10_H_13_N_3_O_3_S·2H_2_O	*F*(000) = 308
*M**_r_* = 291.33	*D*_x_ = 1.490 Mg m^−^^3^
Monoclinic, *P*2_1_	Mo *K*α radiation, λ = 0.71073 Å
Hall symbol: P 2yb	Cell parameters from 3449 reflections
*a* = 4.6645 (4) Å	θ = 2.5–28.2°
*b* = 10.4006 (9) Å	µ = 0.27 mm^−^^1^
*c* = 13.5381 (11) Å	*T* = 100 K
β = 98.674 (1)°	Prism, yellow
*V* = 649.27 (10) Å^3^	0.30 × 0.20 × 0.10 mm
*Z* = 2	

### Data collection

**Table:**

Bruker SMART APEX diffractometer	2937 independent reflections
Radiation source: fine-focus sealed tube	2826 reflections with *I* > 2σ(*I*)
graphite	*R*_int_ = 0.025
ω scans	θ_max_ = 27.5°, θ_min_ = 1.5°
Absorption correction: multi-scan (*SADABS*; Sheldrick, 1996)	*h* = −6→5
*T*_min_ = 0.923, *T*_max_ = 0.973	*k* = −13→13
6232 measured reflections	*l* = −17→17

### Refinement

**Table:**

Refinement on *F*^2^	Secondary atom site location: difference Fourier map
Least-squares matrix: full	Hydrogen site location: inferred from neighbouring sites
*R*[*F*^2^ > 2σ(*F*^2^)] = 0.028	H atoms treated by a mixture of independent and constrained refinement
*wR*(*F*^2^) = 0.074	*w* = 1/[σ^2^(*F*_o_^2^) + (0.0442*P*)^2^ + 0.0466*P*] where *P* = (*F*_o_^2^ + 2*F*_c_^2^)/3
*S* = 1.03	(Δ/σ)_max_ = 0.001
2937 reflections	Δρ_max_ = 0.24 e Å^−^^3^
208 parameters	Δρ_min_ = −0.19 e Å^−^^3^
12 restraints	Absolute structure: Flack (1983), 1370 Friedel pairs
Primary atom site location: structure-invariant direct methods	Flack parameter: −0.05 (6)

### Fractional atomic coordinates and isotropic or equivalent isotropic displacement parameters (Å^2^)

**Table:**

	*x*	*y*	*z*	*U*_iso_*/*U*_eq_	
S1	1.52160 (9)	1.00002 (4)	0.04613 (3)	0.01642 (10)	
O1	1.0480 (3)	0.82234 (12)	0.44254 (9)	0.0175 (3)	
O2	0.4114 (3)	0.51122 (13)	0.53548 (9)	0.0185 (3)	
O3	0.5005 (3)	0.56687 (12)	0.18897 (9)	0.0171 (3)	
O1W	0.0357 (3)	0.42702 (13)	0.15044 (10)	0.0204 (3)	
O2W	0.0077 (3)	0.21977 (12)	0.01907 (10)	0.0197 (3)	
N1	1.1216 (3)	0.83376 (14)	0.24708 (10)	0.0136 (3)	
N2	1.2187 (3)	0.87300 (14)	0.16047 (10)	0.0139 (3)	
N3	1.5378 (3)	1.01558 (15)	0.24590 (10)	0.0147 (3)	
C1	0.8575 (3)	0.72575 (16)	0.41274 (13)	0.0133 (3)	
C2	0.7317 (4)	0.66456 (16)	0.48651 (13)	0.0152 (3)	
H2	0.7859	0.6873	0.5547	0.018*	
C3	0.5256 (4)	0.56959 (17)	0.45936 (12)	0.0149 (3)	
C4	0.4389 (4)	0.53585 (15)	0.36022 (12)	0.0142 (3)	
H4	0.2932	0.4726	0.3427	0.017*	
C5	0.5697 (4)	0.59662 (15)	0.28686 (13)	0.0136 (3)	
C6	0.7848 (4)	0.69196 (15)	0.31126 (13)	0.0126 (3)	
C7	0.9158 (4)	0.74927 (16)	0.23201 (13)	0.0135 (3)	
H7	0.8477	0.7237	0.1652	0.016*	
C8	1.4257 (3)	0.96307 (15)	0.15970 (13)	0.0131 (3)	
C9	1.7695 (4)	1.11131 (17)	0.25974 (13)	0.0179 (4)	
H9A	1.8548	1.1188	0.1973	0.021*	
H9B	1.9242	1.0822	0.3133	0.021*	
C10	1.6620 (5)	1.24156 (18)	0.28688 (17)	0.0274 (4)	
H10A	1.8236	1.3028	0.2953	0.041*	
H10B	1.5812	1.2350	0.3495	0.041*	
H10C	1.5113	1.2715	0.2335	0.041*	
H1O	1.132 (4)	0.846 (2)	0.3945 (12)	0.022 (6)*	
H2O	0.280 (4)	0.462 (2)	0.5106 (17)	0.036 (7)*	
H3O	0.356 (4)	0.517 (2)	0.1785 (17)	0.029 (6)*	
H11	0.024 (5)	0.3659 (19)	0.1092 (18)	0.048 (8)*	
H12	−0.122 (4)	0.467 (2)	0.1412 (19)	0.059 (10)*	
H21	−0.127 (3)	0.1711 (18)	0.0304 (18)	0.029 (7)*	
H22	0.162 (3)	0.178 (2)	0.022 (2)	0.047 (8)*	
H2N	1.140 (4)	0.841 (2)	0.1046 (10)	0.020 (5)*	
H3N	1.476 (4)	0.9852 (19)	0.2978 (10)	0.014 (5)*	

### Atomic displacement parameters (Å^2^)

**Table:**

	*U*^11^	*U*^22^	*U*^33^	*U*^12^	*U*^13^	*U*^23^
S1	0.01776 (19)	0.0203 (2)	0.01189 (19)	−0.00408 (18)	0.00442 (14)	0.00107 (17)
O1	0.0181 (6)	0.0191 (6)	0.0151 (6)	−0.0046 (5)	0.0025 (5)	−0.0017 (5)
O2	0.0223 (6)	0.0215 (6)	0.0122 (6)	−0.0044 (6)	0.0042 (5)	0.0020 (5)
O3	0.0195 (6)	0.0208 (6)	0.0113 (6)	−0.0057 (5)	0.0031 (5)	−0.0035 (5)
O1W	0.0173 (7)	0.0228 (7)	0.0204 (7)	−0.0006 (5)	0.0009 (5)	−0.0031 (5)
O2W	0.0190 (7)	0.0185 (7)	0.0215 (7)	−0.0023 (6)	0.0033 (6)	0.0019 (5)
N1	0.0141 (7)	0.0146 (6)	0.0128 (7)	0.0017 (5)	0.0042 (6)	0.0024 (5)
N2	0.0155 (7)	0.0167 (7)	0.0097 (7)	−0.0028 (5)	0.0029 (6)	0.0004 (5)
N3	0.0158 (7)	0.0176 (7)	0.0117 (6)	−0.0026 (6)	0.0052 (5)	−0.0001 (6)
C1	0.0114 (8)	0.0134 (7)	0.0150 (8)	0.0024 (6)	0.0014 (6)	−0.0002 (6)
C2	0.0165 (9)	0.0182 (8)	0.0102 (8)	0.0027 (7)	0.0001 (6)	−0.0001 (6)
C3	0.0168 (8)	0.0146 (8)	0.0142 (8)	0.0038 (7)	0.0057 (6)	0.0033 (6)
C4	0.0139 (8)	0.0136 (8)	0.0153 (8)	−0.0008 (6)	0.0033 (6)	−0.0008 (6)
C5	0.0141 (8)	0.0137 (7)	0.0133 (8)	0.0026 (6)	0.0033 (6)	0.0004 (6)
C6	0.0115 (8)	0.0129 (7)	0.0137 (8)	0.0025 (6)	0.0027 (6)	0.0011 (6)
C7	0.0150 (8)	0.0141 (8)	0.0115 (8)	0.0028 (6)	0.0020 (6)	0.0000 (6)
C8	0.0118 (8)	0.0134 (7)	0.0146 (8)	0.0019 (6)	0.0033 (6)	0.0020 (6)
C9	0.0164 (9)	0.0202 (8)	0.0171 (9)	−0.0032 (7)	0.0031 (7)	−0.0027 (7)
C10	0.0308 (11)	0.0174 (9)	0.0363 (12)	−0.0024 (8)	0.0122 (9)	0.0012 (8)

### Geometric parameters (Å, °)

**Table:**

S1—C8	1.7085 (17)	N3—H3N	0.86 (1)
O1—C1	1.362 (2)	C1—C2	1.387 (2)
O1—H1O	0.85 (1)	C1—C6	1.409 (2)
O2—C3	1.3710 (19)	C2—C3	1.389 (2)
O2—H2O	0.83 (1)	C2—H2	0.9500
O3—C5	1.352 (2)	C3—C4	1.387 (2)
O3—H3O	0.85 (1)	C4—C5	1.393 (2)
O1W—H11	0.84 (1)	C4—H4	0.9500
O1W—H12	0.84 (1)	C5—C6	1.414 (2)
O2W—H21	0.84 (1)	C6—C7	1.442 (2)
O2W—H22	0.84 (1)	C7—H7	0.9500
N1—C7	1.294 (2)	C9—C10	1.509 (3)
N1—N2	1.3809 (19)	C9—H9A	0.9900
N2—C8	1.346 (2)	C9—H9B	0.9900
N2—H2N	0.86 (1)	C10—H10A	0.9800
N3—C8	1.323 (2)	C10—H10B	0.9800
N3—C9	1.461 (2)	C10—H10C	0.9800
			
C1—O1—H1O	110.3 (16)	O3—C5—C4	121.98 (15)
C3—O2—H2O	108.4 (17)	O3—C5—C6	116.47 (15)
C5—O3—H3O	111.8 (16)	C4—C5—C6	121.55 (15)
H11—O1W—H12	107.8 (15)	C1—C6—C5	117.44 (15)
H21—O2W—H22	109.8 (15)	C1—C6—C7	123.77 (15)
C7—N1—N2	113.40 (14)	C5—C6—C7	118.80 (15)
C8—N2—N1	122.67 (15)	N1—C7—C6	123.43 (15)
C8—N2—H2N	118.4 (15)	N1—C7—H7	118.3
N1—N2—H2N	118.9 (15)	C6—C7—H7	118.3
C8—N3—C9	125.54 (14)	N3—C8—N2	117.97 (15)
C8—N3—H3N	115.7 (14)	N3—C8—S1	125.35 (13)
C9—N3—H3N	118.6 (14)	N2—C8—S1	116.68 (13)
O1—C1—C2	116.95 (15)	N3—C9—C10	112.11 (15)
O1—C1—C6	121.56 (15)	N3—C9—H9A	109.2
C2—C1—C6	121.46 (15)	C10—C9—H9A	109.2
C1—C2—C3	119.14 (15)	N3—C9—H9B	109.2
C1—C2—H2	120.4	C10—C9—H9B	109.2
C3—C2—H2	120.4	H9A—C9—H9B	107.9
O2—C3—C4	121.71 (16)	C9—C10—H10A	109.5
O2—C3—C2	116.61 (15)	C9—C10—H10B	109.5
C4—C3—C2	121.67 (15)	H10A—C10—H10B	109.5
C3—C4—C5	118.68 (16)	C9—C10—H10C	109.5
C3—C4—H4	120.7	H10A—C10—H10C	109.5
C5—C4—H4	120.7	H10B—C10—H10C	109.5
			
C7—N1—N2—C8	178.00 (15)	O3—C5—C6—C1	179.15 (15)
O1—C1—C2—C3	176.95 (15)	C4—C5—C6—C1	−1.5 (2)
C6—C1—C2—C3	−1.2 (2)	O3—C5—C6—C7	−0.6 (2)
C1—C2—C3—O2	179.17 (15)	C4—C5—C6—C7	178.74 (15)
C1—C2—C3—C4	−1.1 (2)	N2—N1—C7—C6	178.51 (14)
O2—C3—C4—C5	−178.27 (15)	C1—C6—C7—N1	2.9 (3)
C2—C3—C4—C5	2.0 (2)	C5—C6—C7—N1	−177.42 (15)
C3—C4—C5—O3	178.64 (15)	C9—N3—C8—N2	177.96 (15)
C3—C4—C5—C6	−0.6 (2)	C9—N3—C8—S1	−2.1 (2)
O1—C1—C6—C5	−175.63 (14)	N1—N2—C8—N3	−0.2 (2)
C2—C1—C6—C5	2.5 (2)	N1—N2—C8—S1	179.78 (12)
O1—C1—C6—C7	4.1 (3)	C8—N3—C9—C10	110.57 (19)
C2—C1—C6—C7	−177.80 (15)		

### Hydrogen-bond geometry (Å, °)

**Table:**

*D*—H···*A*	*D*—H	H···*A*	*D*···*A*	*D*—H···*A*
O1—H1o···N1	0.85 (1)	1.99 (2)	2.722 (2)	144 (2)
O2—H2o···O1^i^	0.83 (1)	2.27 (2)	2.955 (2)	140 (2)
O3—H3o···O1W	0.85 (2)	1.76 (2)	2.598 (2)	174 (2)
O1w—H11···O2w	0.84 (1)	1.94 (1)	2.785 (2)	177 (3)
O1w—H12···O3^ii^	0.84 (1)	2.22 (1)	3.002 (2)	155 (2)
O2w—H21···S1^iii^	0.84 (1)	2.45 (1)	3.279 (1)	169 (2)
O2w—H22···S1^iv^	0.84 (1)	2.49 (1)	3.292 (1)	162 (2)
N2—H2n···O2w^v^	0.86 (1)	2.13 (1)	2.965 (2)	166 (2)
N3—H3n···O2^vi^	0.86 (1)	2.26 (2)	2.934 (2)	136 (2)

Symmetry codes: (i) −*x*+1, *y*−1/2, −*z*+1; (ii) *x*−1, *y*, *z*; (iii) *x*−2, *y*−1, *z*; (iv) *x*−1, *y*−1, *z*; (v) −*x*+1, *y*+1/2, −*z*; (vi) −*x*+2, *y*+1/2, −*z*+1.
